# Complete mitochondrial genomes of *Notropis oxyrhynchus* and *Notropis buccula* (Cypriniformes: Leuciscidae)

**DOI:** 10.1080/23802359.2024.2429632

**Published:** 2024-11-19

**Authors:** A. T. Fields, K. W. Conway, E. P. Dolan, D. G. Swift, A. A. Monroe, C. M. Hollenbeck, P. T. Bean, J. D. Anderson, D. S. Portnoy

**Affiliations:** a Marine Genomics Laboratory, Department of Life Sciences, Texas A&M University–Corpus Christi, Corpus Christi, TX, USA; b Department of Ecology and Conservation Biology and Biodiversity Research and Teaching Collections, Texas A&M University, College Station, TX, USA; c Heart of the Hills Fisheries Science Center, Texas Parks and Wildlife Department, Inland Fisheries, TX, USA; d Perry R. Bass Marine Fisheries Research Station and Hatchery, Texas Parks and Wildlife Department, Coastal Fisheries, Palacios, TX, USA

**Keywords:** Sharpnose shiner, smalleye shiner, mitochondrial genome, Brazos River, Cypriniformes, Leuciscidae

## Abstract

The Leuciscidae (minnows, shiners and relatives) is a diverse family of freshwater fishes with many species endangered due to anthropogenic stressors. *Notropis oxyrhynchus* and *Notropis buccula* are two shiners found only in the upper Brazos River basin in Texas, USA and listed as endangered due to contracted habitat. The complete mitochondrial genome was sequenced for two vouchered specimens for each species; *Notropis oxyrhynchus* having a total mitogenome length of 16,711 bp and *N. buccula* having a total mitogenome length 16685–16686 bp, with both including 13 protein-coding genes, 22 transfer RNAs genes, and 2 ribosomal RNA genes. Phylogenetic analysis supports previous hypotheses regarding placement of these species.

## Introduction

**Figure 1.**
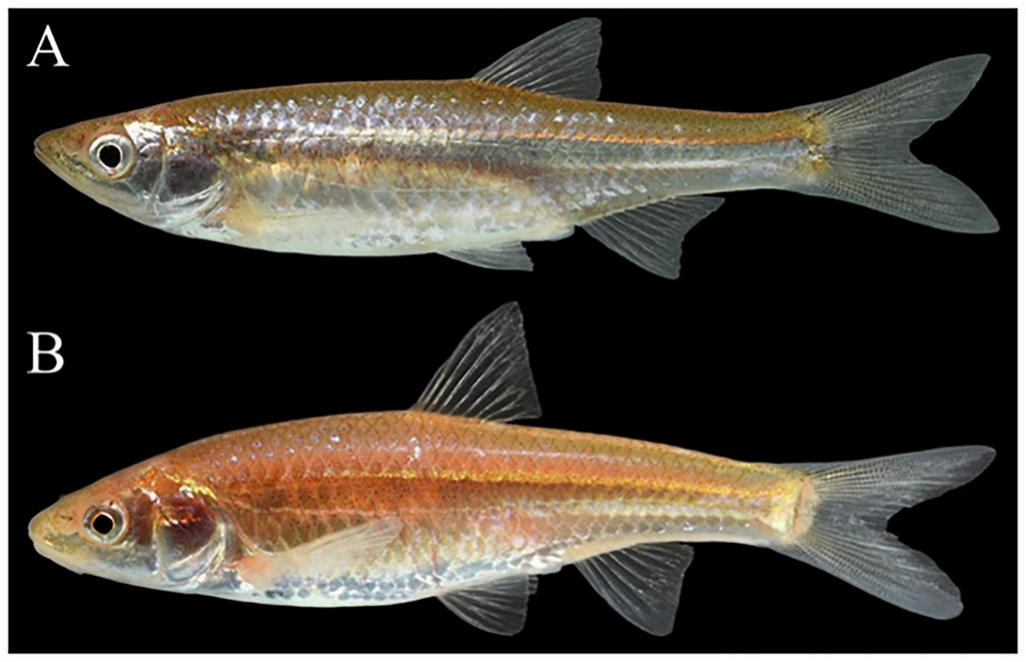
*Notropis oxyrhynchus* TCWC 20309.01 (A) and *N. buccula* TCWC 20309.02 (B) stored at the Texas A&M University Biodiversity Research and Teaching Collections. Photos by K.W. Conway.

Many freshwater fishes are imperiled due to population fragmentation and anthropogenic habitat alteration, including increased climate variability (Jelks et al. 2008). Small-bodied species with short lifecycles living near the edge of their environmental tolerance within arid or semi-arid regions are especially vulnerable (Chessman 2013). *Notropis oxyrhynchus* (Hubbs and Bonham 1951) and *Notropis buccula* (Cross 1953) (Figure 1) are two such examples from the North American genus *Notropis* (Leuciscidae), that are restricted to the upper Brazos River basin in semi-arid regions of Texas, United States (US). Reduced flow and river fragmentation have limited reproductive success in these short-lived species (Wilde and Urbanczyk 2013), which require high flow events for successful spawning and recruitment (Worthington et al. 2018). Due to a combination of factors, *N. oxyrhynchus* and *N. buccula* were listed under the U.S. Endangered Species Act in 2014 (National Archives 2013) and are also considered endangered by Texas Parks and Wildlife Department as well as Vulnerable by the IUCN Red List (NatureServe, 2013a,b), spurring a need to better understand these imperiled species. However, genetic resources for both species are scarce as no sequence data is available for *N. buccula* and only one cytochrome B sequence is available for *N. oxyrhynchus*. Therefore, mitochondrial genomes provide a much-needed resource for research and management.

## Materials and methods

### Sample collection

Specimens were collected from the upper Brazos River at four locations within Baylor and Knox counties (Table 1), identified to the level of species using taxonomic keys available in Hubbs et al. (2008) and species identification corroborated using original taxonomic descriptions (Hubbs and Bonham 1951; Cross 1953). After collection, specimens were photographed using a Nikon D850 and euthanized *via* an overdose of eugenol. After euthanasia, the right pectoral fin, the right pelvic fin and a muscle sample were dissected from photographed individuals and preserved in 100% ethanol. Euthanized specimens were fixed in 10% neutral buffered formalin (with subsequent transfer to 70% ethanol) or preserved in 100% ethanol, and deposited at the Texas A&M University Biodiversity Research and Teaching Collections (TCWC, Kevin W. Conway, kevin.conway@tamu.edu).

**Table 1.** Museum voucher number and collection details for the specimens used in this study.

Voucher number	Genus	Species	County	Water Body	Latitude	Longitude	Date collected	Average Coverage depth
TCWC 20350.02	*Notropis*	*oxyrhynchus*	Knox	Brazos River	33.55867	−99.5112	9/25/2021	352
TCWC 20309.01	*Notropis*	*oxyrhynchus*	Baylor	Brazos River	33.58113	−99.2677	5/12/2021	256
TCWC 20310.02	*Notropis*	*buccula*	Knox	Brazos River	33.54850	−99.6599	5/13/2021	160
TCWC 20351.04	*Notropis*	*buccula*	Knox	Brazos River	33.50032	−99.8022	9/25/2021	250

All specimens are archived at the Texas A&M University Biodiversity Research and Teaching Collections (TCWC).

**Figure 2.**
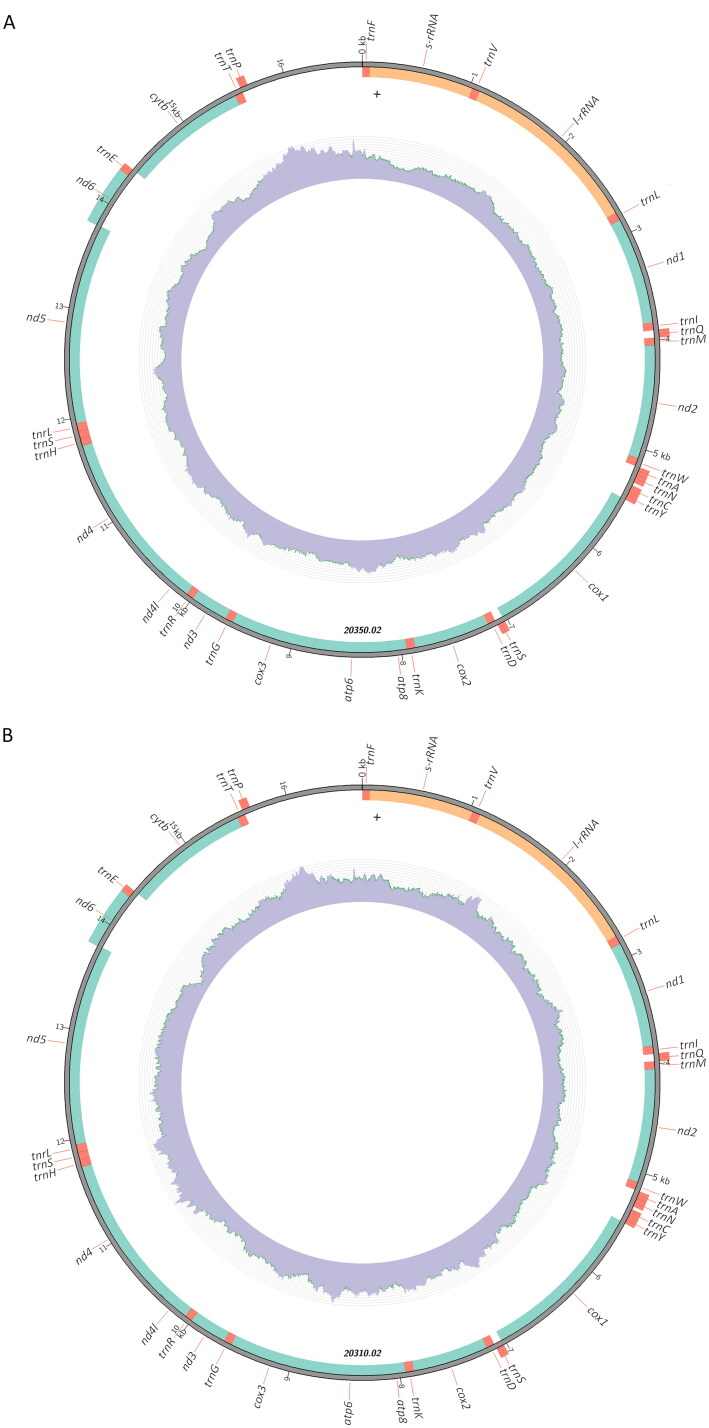
Mitogenome map of (A) *Notropis oxyrhynchus*, vouchered specimen TCWC 20350.02 (GenBank PP467616), and (B) *Notropus buccula*, vouchered specimen TCWC 20310.02 (GenBank PP467618), which contain the same genes and gene order as all the specimens sequenced. The outer map denotes heavy strand coding sequences on the outside and the light strand coding sequences on the inside. The internal ring denotes the sequencing coverage depth.

DNA was extracted from muscle samples using Mag-Bind Blood and Tissue DNA Kit (Omega Bio-tek, Norcross, GA) and the whole genome sequencing (WGS) library was prepared following a modified version of Jones et al. (2023) using the Illumina DNA prep kit (Illumina, San Diego, CA) and 0.1 µM of each primer in the PCR step, rather than the original 0.2 µM, as well as Phusion polymerase (New England Biolabs, Ipswitch, MA) instead of Q5 DNA polymerase. The average fragment size was determined with a fragment analyzer (Agilent, Santa Clara, CA) and samples were pooled in equal molar concentration for sequencing on a partial lane of a NovaSeq 6000 (paired end 150 bp; Illumina, San Diego, CA) at Azenta (South Plainfield, NJ). The resulting sequence depth was between 160 to 352 (Table 1; Figure 2).

### Assembly and annotation

The software MitoZ v3.6 (Meng et al. 2019) was used to trim, filter, and create a *de novo* assembly with annotations, from the Illumina reads, using the default settings and associated software (Gertz et al. 2006; Krzywinski et al. 2009; Li and Durbin 2009; Li et al. 2009; Wheeler and Eddy 2013; Li et al. 2015; Huerta-Cepas et al. 2016; Chen et al. 2018; Karlicki et al. 2022). One of the *N. oxyrhynchus* samples (PP467617) did not properly assemble with MitoZ and therefore was mapped to the PP467616 assembly using bwa v 0.7.17 (Li and Durbin 2009) and assembled from mapped reads using samtools v 1.13 (Danecek at al. 2021). This assembly was then annotated with MitoZ.

### Phylogenetic analysis

*Notropis* is a genus of 92 species and is currently under revision because multiple studies have shown that the genus is not monophyletic (Mayden et al. 2006; Schönhuth et al. 2018; Stout et al. 2022). Mayden et al. (2006) and Stout et al. (2022) have suggested that *N. oxyrhynchus* is part of a clade including the ‘true’ *Notropis* and thereby this genus would retain the name *Notropis*, while *N. buccula* is in a clade with several other species of *Notropis* that will likely be placed in the genus *Alburnops*. Given that the two species in this study are not recovered within the same clade, two separate phylogenetic reconstructions (one for each species) are presented. Complete mitochondrial genomes from appropriate congeners were downloaded from GenBank and aligned with MAFFT v 7.525 (Katoh and Standley 2013) separately for each species. IQ-tree v 2.2.6 (Minh et al. 2020) was used to perform a maximum likelihood analysis of the sequences with 1,000 bootstraps. *Notropis volucellus* was used as the outgroup in both analyses, given that it is outside of both clades.

## Results

A mitochondrial genome was assembled for each *N. oxyrhynchus* individual and both were found to be 16,711 bp long. They both included all 37 commonly found genes including 13 protein-coding genes (PCG), 22 transfer RNA (tRNA) genes and two ribosomal RNA (rRNA) genes in addition to the control region (Figure 2A). They also consisted of 27% adenine, 27% cytosine, 27% guanine and 19% thymine, resulting in a 46% GC. All of the PCGs except cytochrome oxidase subunit 1 (*cox1*) began with an ATG start codon while *cox1* began with a GTG start codon. Only seven of the PCGs ended in a traditional stop codon with four ending in T (*nd2*, *cox2*, *nd3* and *cytb*) and two ending in TA (*cox3*, *nd4*). All of the rRNAs and PCGs were on the light strand except NADH dehydrogenase subunit 6 (*nd6*) and eight of the tRNAs which were on the heavy strand.

A mitochondrial genome was assembled for each *N. buccula* individual and there was a minor sequence length variation between the *N. buccula* samples as PP467618 was 16,685 bp long while PP467619 was 16,686 bp long. Both included all 37 commonly found genes including 13 PCGs, 22 tRNA genes and two rRNA genes in addition to the control region (Figure 2B). They also consist of 28% adenine, 27% cytosine, 27% guanine and 18% thymine which resulted in a 45% GC. All the PCGs except cytochrome oxidase subunit 1 (*cox1*) began with an ATG start codon while *cox1* began with a GTG start codon. Only seven of the PCGs ended in a traditional stop codon with four ending in T (*nd2*, *cox2*, *nd3* and *cytb*) and two ending in TA (*cox3*, *nd4*). All the rRNAs and PCGs were on the light strand except nd6 and eight of the tRNAs which were on the heavy strand. The length variation between the two *N. buccula* genome sequences includes indels in two genes. In NADH dehydrogenase subunit 3 (*nd3*), a triple thymine indel was found which codes for an extra phenylalanine residue in PP467618 and PP467619 was 2 bp longer in the 16S ribosomal RNA and 2 bp longer in the control region.

**Figure 3.**
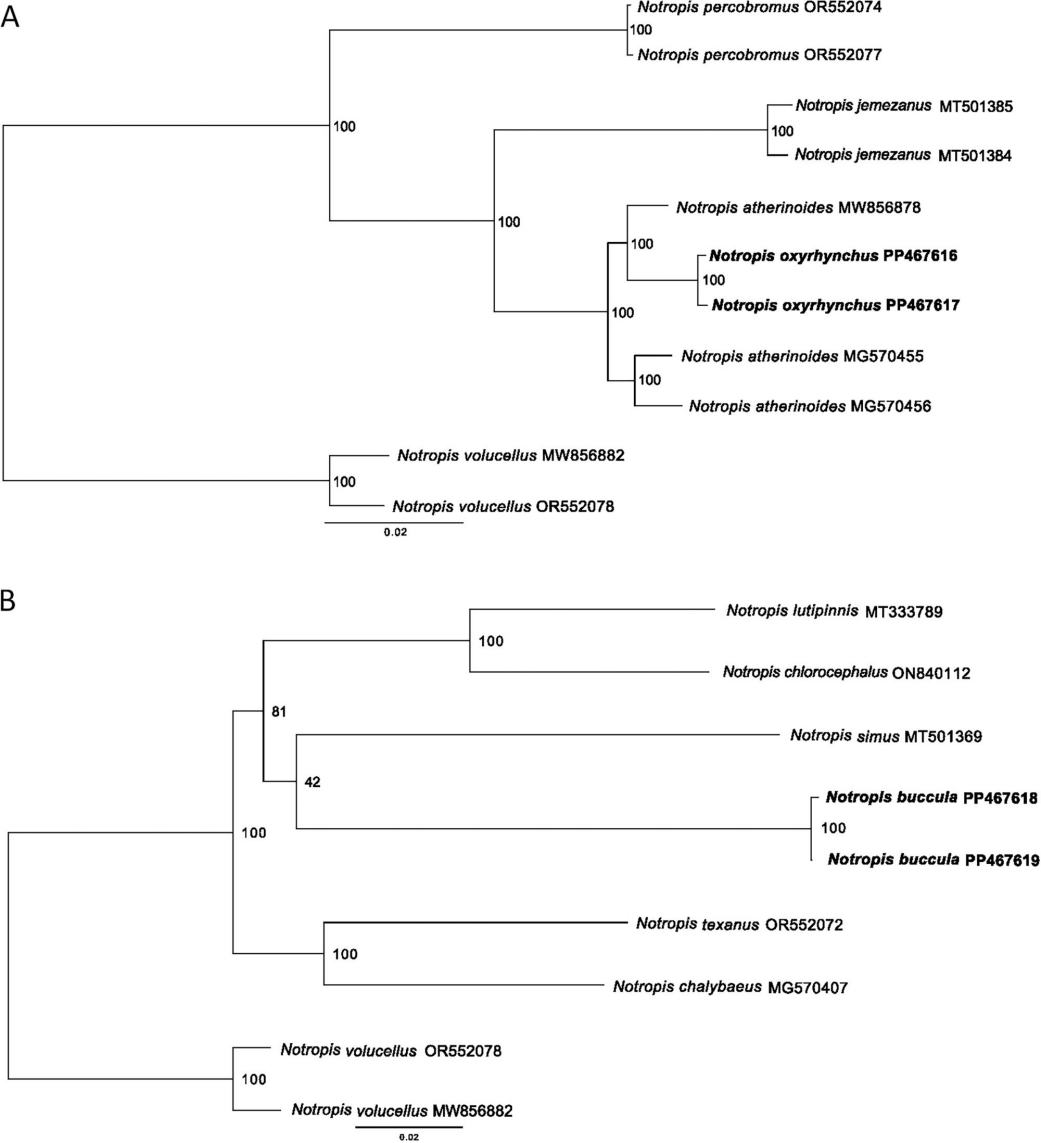
Maximum likelihood trees of the two *Notropis* groups including the (A) ‘true’ *Notropis* species and the (B) *Alburnops* associated species. *Notropis oxyrhynchus* PP467616-PP467617 (this study) were compared with the complete mitogenomes of *Notropis percobromus* OR552074 (Lee et al. 2024), *Notropis percobromus* OR552077 (Lee et al. 2024), *Notropis jemezanus* MT501385 (Diver et al. 2024), *Notropis jemezanus* MT501384 (Diver et al. 2024), *Notropis atherinoides* MW856878, *Notropis atherinoides* MG570455 (Schroeter et al. 2020) and *Notropis atherinoides* MG570456 (Schroeter et al. 2020) which were downloaded from GenBank. *Notropis buccula* PP467618-PP467619 (this study) were compared with the complete mitogenomes of *Notropis lutipinnis* MT333789 (Bobier 2020), *Notropis chlorocephalus* ON840112, *Notropis simus pecosensis* MT501369 (Diver et al. 2024), *Notropis texanus* OR552072 (Lee et al. 2024) and *Notropis chalybaeus* MG570407 (Schroeter et al. 2020) which were downloaded from GenBank. *Notropis volucellus* MW856882 and *Notropis volucellus* OR552078 (Lee et al. 2024) were used as outgroups in both analyses. Sequences generated in this study are in bold and bootstrap support values are denoted at the nodes.

Phylogenetic analysis of available mitochondrial genomes of ‘true’ *Notropis* related to *N. oxyrhynchus*, using the optimal mutation model selected (TIM3 + F + I + G4), found that *N. oxyrhynchus* groups most closely to *N. atherinoides* (Figure 3). Both *N. oxyrhynchus* sequences were sister to each other and found nested inside of the *N. atherinoides* clade. The genetic distance within *N. oxyrhynchus* is smaller (approximately 10 times) than the distance between *N. oxyrhynchus* and the *N. atherinoides* from New York (MG570455.1, MG570456.1) and southern Wisconsin (AP012083.1; Table 2). The distance between the northern Wisconsin *N. atherinoides* (MW856878.1) and the other *N. atherinoides* is similar to the distance between the northern Wisconsin sample and *N. oxyrhynchus* (Table 2). This clade is also distinct from its sister species, *N. jemezanus* (Figure 3).

**Table 2.** The genetic distance between *N. oxyrhynchus* and *N. antherinoides* samples calculated using a TIM3 mutation model.

Catch location	Sample	*N. oxyrhynchus* PP467616	*N. oxyrhynchus* PP467617	*N. atherinoides* MW856878	*N. atherinoides* AP012083	*N. atherinoides* MG570455	*N. atherinoides* MG570456
Texas,Brazos River	*N. oxyrhynchus* PP467616	–	0.0025	0.0172	0.0239	0.0236	0.0240
Texas,Brazos River	*N. oxyrhynchus* PP467617	0.0025	–	0.0175	0.0243	0.0239	0.0242
Wisconsin, Lake Superior	*N. atherinoides* MW856878	0.0172	0.0175	–	0.0185	0.0174	0.0190
Wisconsin, Waupaca	*N. atherinoides* AP012083	0.0239	0.0243	0.0185	–	0.0117	0.0030
New York	*N. atherinoides* MG570455	0.0236	0.0239	0.0174	0.0117	–	0.0121
New York	*N. atherinoides* MG570456	0.0240	0.0242	0.0190	0.0030	0.0121	–

Phylogenetic analysis of the *N. buccula* and other available mitochondrial genomes of species considered to belong to *Alburnops* was performed using the optimal mutation model selected (TIM2 + F + G4). *Notropis buccula* was found to be sister to another endangered southwestern shiner, *N. simus*. These two species are sister to *N. lutipinnis* and *N. chlorocephalus*.

## Discussion and conclusion

This study is the first to sequence and characterize the mitochondrial genome of *N. oxyrhynchus* and *N. buccula*. The four new mitogenomes have the same genes, gene arrangements and similar lengths to those available for other *Notropis* species.

This work supports previous phylogenetic hypotheses for a sister group relationship between *N. oxyrhynchus* and *N. atherinoides* (Snelson 1968; Bielawski and Gold 2001; Stout et al. 2022). However, while Bielawski and Gold (2001) studied the *N. atherinoides* clade, they did not report the nesting of *N. oxyrhynchus* within *N. atherinoides* reported here. Given the paraphyly of *N. atherinoides* and genetic distance between the northern Wisconsin *N. atherinoides* to the other *N. atherinoides* and *N. oxyrhynchus*, there may be cryptic diversity within *N. atherinoides* (as suggested by April et al. 2011), which warrants further study of this species.

Our phylogenetic analyses also support the hypothesis that *Notropis buccula* is a close relative of species considered to belong to the genus *Alburnops* (Mayden et al. 2006; Stout et al. 2022), though mitogenomes are currently unavailable for several of the putative members of this genus as proposed by Stout et al. (2022). While not a rigorous test of these hypotheses, our work suggests that further analysis is warranted to better understand the evolutionary history of *Notropis* as well as the limits of some currently recognized species and genera (i.e. Stout et al. 2022).

## Data Availability

The data that support the findings of this study are openly available in NCBI at https://www.ncbi.nlm.nih.gov (GenBank: PP467616–PP467619, BioProject: PRJNA1135998, BioSample: SAMN42498145–SAMN42498148, SRA: SRX25330954–SRX25330957).
